# A Validated Genome Wide Association Study to Breed Cattle Adapted to an Environment Altered by Climate Change

**DOI:** 10.1371/journal.pone.0006676

**Published:** 2009-08-18

**Authors:** Ben J. Hayes, Phil J. Bowman, Amanda J. Chamberlain, Keith Savin, Curt P. van Tassell, Tad S. Sonstegard, Mike E. Goddard

**Affiliations:** 1 Biosciences Research Division, Department of Primary Industries Victoria, Melbourne, Victoria, Australia; 2 United States Department of Agriculture, Agricultural Research Service, Bovine Functional Genomics Laboratory, Beltsville, Maryland, United States of America; 3 Faculty of Land and Food Resources, University of Melbourne, Melbourne, Victoria, Australia; Universidade de Vigo, Spain

## Abstract

Continued production of food in areas predicted to be most affected by climate change, such as dairy farming regions of Australia, will be a major challenge in coming decades. Along with rising temperatures and water shortages, scarcity of inputs such as high energy feeds is predicted. With the motivation of selecting cattle adapted to these changing environments, we conducted a genome wide association study to detect DNA markers (single nucleotide polymorphisms) associated with the sensitivity of milk production to environmental conditions. To do this we combined historical milk production and weather records with dense marker genotypes on dairy sires with many daughters milking across a wide range of production environments in Australia. Markers associated with sensitivity of milk production to feeding level and sensitivity of milk production to temperature humidity index on chromosome nine and twenty nine respectively were validated in two independent populations, one a different breed of cattle. As the extent of linkage disequilibrium across cattle breeds is limited, the underlying causative mutations have been mapped to a small genomic interval containing two promising candidate genes. The validated marker panels we have reported here will aid selection for high milk production under anticipated climate change scenarios, for example selection of sires whose daughters will be most productive at low levels of feeding.

## Introduction

Likely effects of climate change are rising temperatures in some food production areas, water shortages and rising grain prices due to increased demand for human food and biofuel feedstuffs [1]–[7]. As a result, future dairy farming systems may become increasingly reliant on pasture instead of grain to feed cows. In this scenario, the selection of dairy cows that can produce at high levels with lower levels of feeding is important. As cattle reproduce slowly, we need to develop methods to select suitable cattle before the change in production systems occurs. Fortunately the range of production environments in which dairying is already carried out in Australia is wide, from fully pasture based systems to fully feedlot based systems, and from tropical climate to temperate climate [8]. This gives us a chance to discover loci or genetic markers that can be used to select cattle that are suitable for future farming systems.

Genetic variation in the sensitivity of milk production of dairy cows to environment has been reported. For example, as heat stress is increased, dairy sires change ranking in their estimated breeding values (EBVs) for milk yield [8]–[9]. Some re-ranking of dairy sires based on the level of feeding of their daughters has also been reported [8], [10]. This indicates that it is possible to select cows that are less sensitive to heat stress and low feeding level than average cows. Although traditional selection methods could be used to achieve this change in environmental sensitivity, gains could be accelerated if the loci responsible for the genotype by environment interaction, or DNA markers in linkage disequilibrium with these loci, could be identified and then used in marker assisted selection. In an attempt to find such genetic markers, we combine milk production recording information and historical climatic data from a wide range of environments across Australia, with genome wide dense single nucleotide polymorphism (SNP) data on dairy sires. This enabled a genome wide association study for sensitivity of milk production to environmental parameters. An across breed validation strategy was used to refine the genomic interval containing the causative mutation underlying these associations.

## Methods

**Figure 1:**
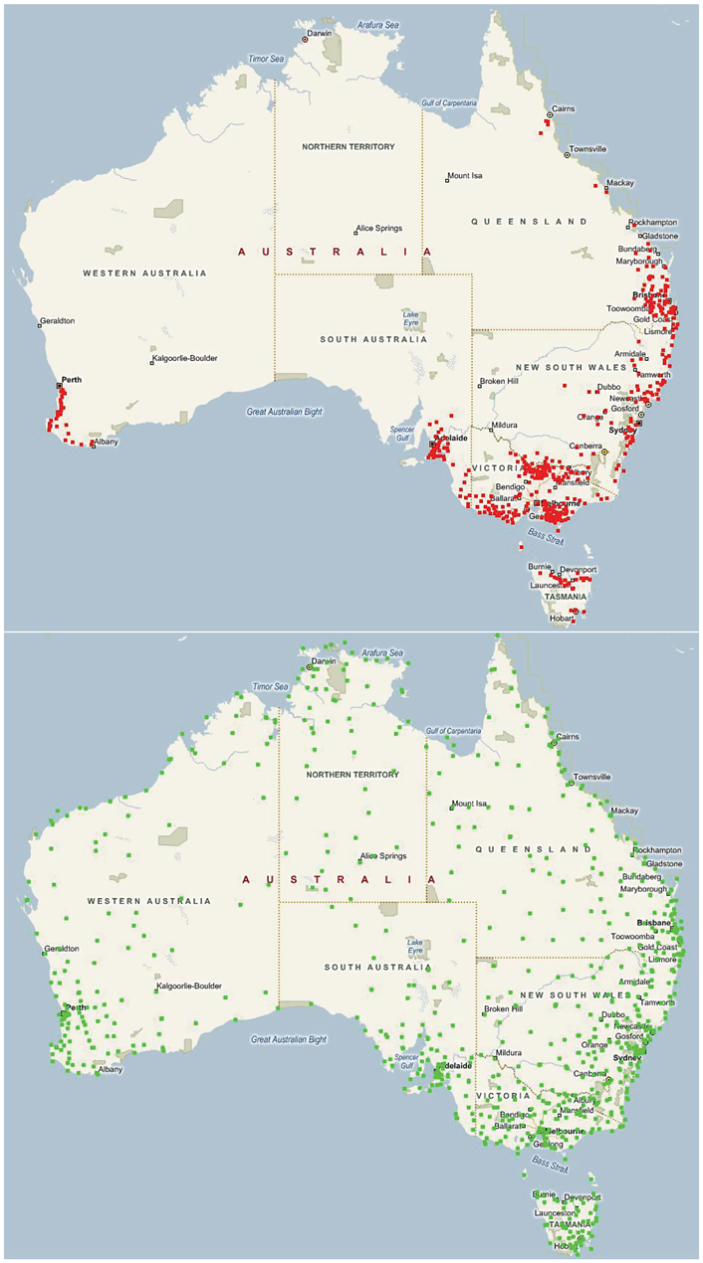
Location of dairy farms for which milk production data was retrieved and location of weather stations providing climate data. A. Location of dairy farms and B. Location of weather stations (green pins) supplying data in 2008 to the Australian Bureau of Meteorology (http://www.bom.gov.au/silo) that were in turn used by the Queensland Department of Environment and Resource Management DataDrill project (http://www.longpaddock.qld.gov.au/silo) in interpolating meteorological data onto a 5-×5-km grid across Australia. Image created with using http://www.microsoft.com/maps/isdk/ajax/ .

Three data sets were used. The discovery data set consisted of first lactation test day milk yield records of 62343 Holstein Friesian cows sired by 798 sires, milking across the range of environments of dairying in Australia, from the Australian Dairy Herd Improvement Scheme (ADHIS) database. The first validation data set consisted of first lactation test day milk yield records of 23603 cows sired by a different set of 453 Holstein bulls, none of which sired cows in the discovery data set. The second validation set consisted of first lactation test day records from 35293 Jersey cows, sired by 364 Jersey bulls, a different breed of dairy cattle. Within each data set, the average daily milk production for each herd (herd test day milk yield or HTDMY) at the time the cows were milked was used as a surrogate for the level of feeding, as actual feeing information is unavailable on this scale, and average daily milk production in a herd has a close relationship with actual level of feeding [11]. Temperature and humidity data for each date of test were extracted from a dataset provide by the Queensland Department of Environment and Resource Management DataDrill [12] project. These records are derived from interpolation of meteorological station data onto a 5-×5-km grid across Australia. The data are interpolated onto a two-dimensional spline providing the “best estimate” of daily weather variables on a 5-×5-km grid. The dairy farms in the study were located near a number of meteorological stations recording daily weather measurements, Figure 1. These data were used to calculate the temperature humidity index (THI) on the day of milking for use as a measure of heat stress [8]. In *Bos taurus* cattle, stress as measured by respiration rate increases rapidly when the maximum daily THI is above 74 units [13], [14]. Earlier investigations showed heat stress only affected milk production above 60 THI units [8]. To accommodate this when THI was the environmental descriptor, all values of THI below 60 were given the value of 60.

The next step was to derive the sensitivity of the milk yield of the sires of the cows to changes in either THI or HTDMY, again within each data set. We did this by regressing the sire's daughters daily milk yield on the environmental variable for the same day (THI or HTDMY) using a random regression model The intercept of the regression is the relative average milk production of the sires's daughters at the mean level of the environmental variable. The slope can be interpreted as the sensitivity of the milk yield of a bull's daughters to changes in the environmental variable. These traits are designated HTDMY_int_, HTDMY_slope_, THI_int_ and THI_slope_. The model used to estimate daughter yield deviations for the four traits was 
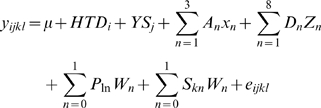
where *y_ijkl_* is yield of milk from the *i* ^th^ herd test day, *j* ^th^ year season of calving, *k* ^th^ sire and *l* ^th^ cow in her first lactation, μ is the overall mean, *HTD_i_* is the effect of the *i* ^th^ herd test day; *YS_j_* is the effect of the *j* ^th^ year season of calving, *x_n_* is the nth-order orthogonal polynomial corresponding to age on day of test, *A_n_* is a fixed regression coefficient of milk on age at test, *Z_n_* is the nth-order polynomial corresponding to days in milk (DIM) at test, *D_n_* is a fixed regression coefficient of milk yield on DIM, *P*_ln_ is a random regression coefficient on the environmental descriptor for the *l* ^th^ cow, *S_kn_* is a random regression coefficient on the environmental descriptor for the *k* ^th^ sire, *W_n_* is either the intercept (n = 0) or slope (n = 1) solution for HTDMY or THI, and *e_ijkl_* is the vector of residual effects.

In the next step, the 798 Holstein Friesian dairy sires of the cows in the discovery data, the 453 Holstein Friesian dairy sires in first validation data set, and the 364 Jersey sires were genotyped with the Illumina BovineSNP50 beadchip containing more than 56,000 SNP assays [15]. Samples were screened for the proportion of missing genotypes, and animals with greater than 10% missing genotypes were removed. The SNPs were included only if they met the following criteria; call rate >90%, minimum allele frequency >5%, and did not have extreme Hardy Weinberg (HWE) χ2 values (>600). The rational here was to remove SNPs where genotype calls were poorly clustered. In our complex pedigree population, actual HWE values can be quite misleading, so we prefer not to remove SNPs with a lower cut off. The many other quality control steps are likely more effective at removing problematic SNPs than HWE scores. Parentage checking was performed, and any genotypes incompatible with pedigree were removed. There were 781, 400 and 362 samples in the discovery data set, first validation data set and second validation data set respectively with greater than 90% of SNPs genotyped, these were used for further analysis. There were 39048 SNPs that satisfied all selection criteria.

The SNPs were ordered by chromosome position using Bovine Genome Build 4.0 (http://www.ncbi.nlm.nih.gov/projects/genome/guide/cow/). The genotypes were then submitted to fastPHASE [16] chromosome by chromosome. The missing genotypes were taken as those filled in by fastPHASE. Accuracy of filling in missing genotypes was assessed by removing known genotypes at every 50^th^ position for 10% of animals on chromosome 26. Imputed genotypes were then compared to the known genotypes. There were 3571 missing genotypes filled in by the fastPHASE program 3525 of which were correct, giving an accuracy of 98.7%. For comparison, an approach which filled in missing genotypes by sampling from a uniform distribution with mean allele frequency gave an accuracy of only 51.1%. Average marker spacing was 66.5 kb. Average LD between adjacent markers, measured by r^2^, was 0.271.

A linear model was fitted to the sires' daughter yield deviations for HTDMY_int_, HTDMY_slope_, THI_int_ and THI_slope_ to determine if the SNPs accounted for any of the between sire variation in these traits. The top–bottom called genotypes were re-coded as 0 for the homozygote of the first alphabetical allele, 1 for the heterozygote, and 2 for the homozygote of the second alphabetical allele. The SNPs were fitted to the sire solutions for intercept and slope for either HTDMYor THI: 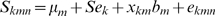 where S_kmn_ is the estimated effect for intercept (m = 0) or slope (m = 1) (analysed separately) for the k^th^ sire with SNP genotype x_km_ (either 0,1 or 2), b_m_ is the effect of the SNP for intercept (m = 0) or slope (m = 1), and Se_k_ is the random residual effect of sire k and other parameters are as defined above. The variance of the sire effects was **A**σ^2^_S_ where **A** is the relationship matrix among the sires and σ^2^_S_ is the sire variance. Fitting the relationship between the sires should remove any spurious associations due to population structure. The relationship was derived from the herdbook pedigree of the sires which dates back to 1940.

All data analyses were performed using mixed linear models with variance components estimated by residual maximum likelihood [17].

## Results and Discussion

**Figure 2:**
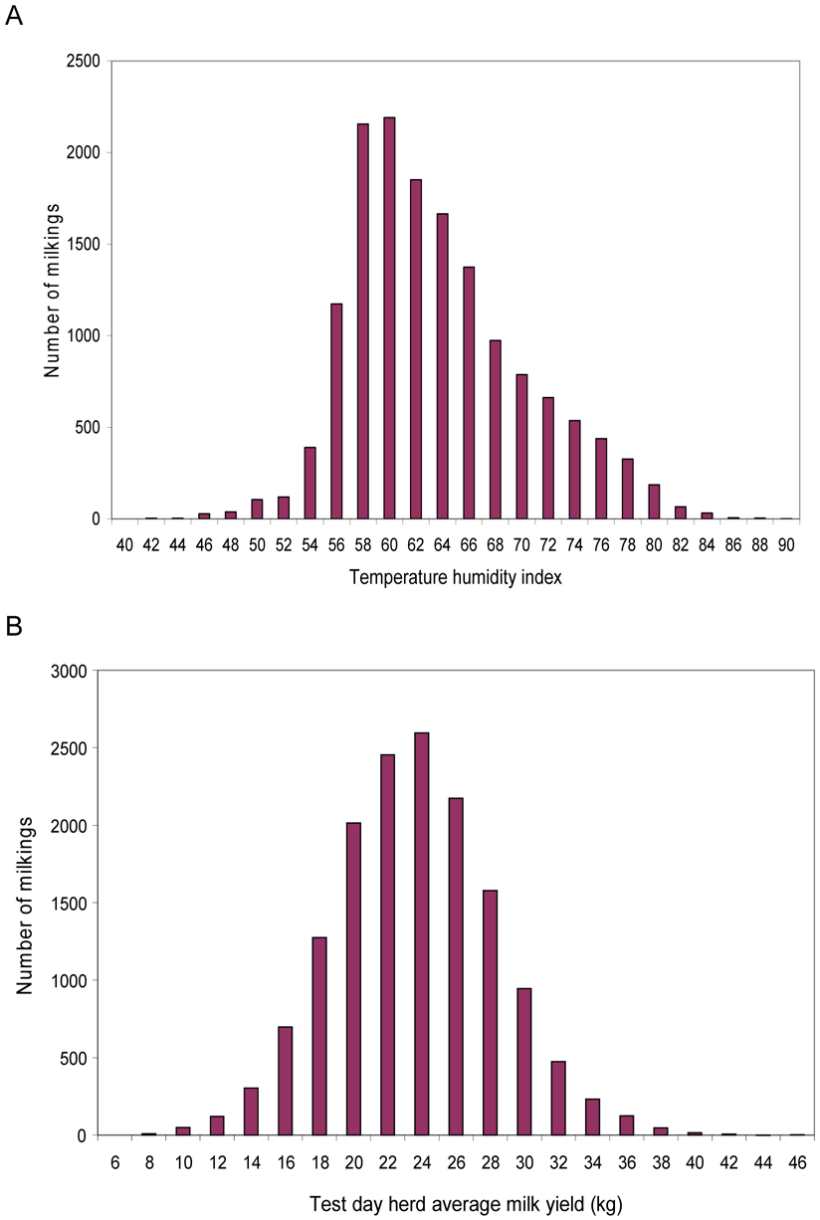
Distribution of environmental variables. A. Distribution of Temperature humidity index (THI) values in the data. B. Distribution of Herd average daily milk yields (HTDMY) in the data.

**Figure 3:**
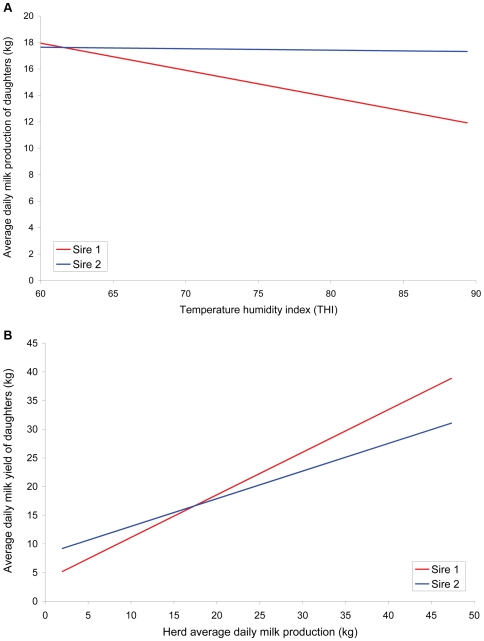
Responses in milk production to environmental variables for different sires A. Predicted response in daily milk production of daughters to temperature humidity index (THI) for the two most extreme sires from the data set. In a climate change scenario where the THI increases significantly, sire 2 should be selected for breeding as the milk yield of his daughters is relatively insensitive to THI. B. Predicted response in daily milk production of daughters to herd average daily milk production (HTDMY), a surrogate for the level of feeding, for two sires from the data set. With low levels of feeding, eg. low inputs of grain, sire 2 could be considered as his daughters produce more milk than the daughters of sire 2 at very low levels of feeding.

The milk production records were sourced from farms across a wide range of environments, Figure 1, with a resulting large range of THI and HTDMY across milk recording days, Figure 2. The results indicated considerable sire by THI and HTDMY interaction, Figure 3. The distributions of both THI and HTDMY in the data set indicated a large range for these environmental descriptors (Figure 2). In a larger data set the genetic correlation between milk production at the 5^th^ and 95^th^ percentile of THI and HTDMY was 0.93 and 0.84 respectively [8].

Using P<0.001 as a significance threshold, a number of significant associations were detected for all four traits (Table 1). False discovery rates (the ratio of expected significant SNPs given the significance level to the actual number of SNPs) were moderate for HTDMY_slope_, and high for THI_slope_. These results are consistent with our previous finding that there is less genetic variation in sensitivity to heat stress than to feeding level [8].

**Table 1:** Number of SNPs significant at P<0.001 by trait and false discovery rates in Holstein Friesian discovery data.

Trait	Number of SNPs significant P<0.001	False discovery rate
HTDMY_int_	152	0.25
HTDMY_slope_	76	0.50
THI_int_	92	0.39
THI_slope_	42	0.91

We then attempted to validate the significant results in the two independent sets of data. The significant SNPs for HTDMY_slope_ and THI_slope_ from the discovery data set were tested in this validation data set. The significant SNPs from the intercept traits are described and discussed in more detail in Pryce et al. [18]. Despite the moderate to high false discovery rate among the SNPs in the discovery data set, more markers were significant (P<0.05) than expected by chance in the validation data set, at least for HTDMY_slope_, Table 2 and 3. For the majority of the validated markers, the direction of the SNP effects was the same in the discovery and Holstein validation data sets, although the magnitude of the estimated effects was generally reduced in the validation data set. For THI_slope_, the number of validated SNPs was lower, however one SNP on BTA29 was validated in both breeds, Table 3.

**Table 2:** Number of significant SNPs validated in the Holstein and Jersey validation sets.

*HTDMY_int_*			
	HD*	HV**	JV***
HD		0.96	−0.34
HV	56		−0.31
JV	20	12	
*HTDMY_slope_*			
	HD	HV	JV
HD	−	0.67	0.69
HV	15	−	1.00
JV	5	1	−
*THI_int_*			
	HD*	HV**	JV***
HD		0.99	−0.02
HV	9		−0.70
JV	11	3	
*THI_slope_*			
	HD*	HV**	JV***
HD	−	1.00	0.88
HV	2	−	−1.00
JV	4	\1	−

The number of SNPs tested in the validation data sets was HTDMY_int_ 152, HTDMY_slope_ 76, THI_int_ 92, THI_slope_ 42, as in Table 2. For each trait, the numbers of SNPs significant in the Holstein Validation (HV) are given in the HV row and Holstein discovery (HD) column, the numbers of SNPs significant in the Jersey Validation (JV) are given in the JV row and HD column, and the number of SNPs validated in both the HV and JV data sets are given in the JV row and the HV column. The numbers on the corresponding upper diagonal are the correlation of SNP solutions for SNPs validated in both data sets.

* HD Holstein discovery.

** HV Holstein Validation.

*** JV Jersey Validation.

**Table 3:** SNPs for HTDMY_slope_ and THI_slope_ validated in the Jersey data set and their effects in the discovery and validation data set.

			F-value			Effect discovery		
Marker Name	Chr	Position	Discovery	Holstein validation	Jersey Validation	Discovery	Holstein validation	Jersey Validation
**HTDMY_slope_**								
*BFGL-NGS-14740	9	13958461	14.0	7.3		−0.42	−0.26	
BTB-02047616	9	32690344	13.5	3.9	6.5	0.43	0.21	0.28
BFGL-NGS-106238	9	33261215	10.9	4.4		−0.39	−0.21	
BTA-112863-no-rs	9	34602197	13.1		5.4	−0.42		−0.31
BTB-00384830	9	35241353	15.2	6.7		−0.47	−0.27	
BFGL-NGS-2259	9	38045164	14.0	9.3		0.43	0.30	
BFGL-NGS-170	9	45345901	14.4	5.3		0.45	0.22	
BFGL-NGS-38561	9	45373250	13.7	4.6		0.42	0.21	
BTB-00402956	9	84338662	11.5	4.7		0.38	0.20	
BFGL-NGS-48434	9	106226558	11.1	4.6		0.38	0.22	
BTB-01568010	11	51716179	13.4		6.2	−0.44		−0.32
Hapmap26998-BTA-152949	13	13253202	11.2		5.0	−0.63		−1.79
BFGL-NGS-64510	13	41797328	14.1	4.4		0.44	0.21	
BTA-71452-no-rs	13	62459428	13.3	6.5		0.43	0.26	
BFGL-NGS-89312	13	62648774	11.0	6.1		0.38	0.25	
BFGL-NGS-20976	13	62755245	14.7	4.0		0.47	0.21	
BTB-00832114	22	4971802	11.3	5.0		0.50	−0.27	
UA-IFASA-6407	22	22434343	11.0		5.1	−0.49		−2.20
Hapmap33956- BES4_Contig493_3026	22	43302073	11.2	5.0		0.38	−0.22	
**THI_slope_**								
BFGL-NGS-139	8	18499827	11.2	8.9		0.16		0.23
BFGL-NGS-89500	10	20564990	11.9	5.4		−0.15	−0.1	
BFGL-BAC-38208	25	32862254	11.4	5.6		−0.12		−0.07
BFGL-NGS-30169	29	48329079	12.1	4.7	5	−0.13	−0.09	0.06

The effects are the result of inheriting one extra copy of the second allele of the SNP (alleles in alphabetical order and called in top-bottom format). The F-value thresholds were 10.84 (P<0.001) for discovery and 3.85 (P<0.05) for validation. Blank cells indicate non-significance.

* Note that the ARS prefix in front of BFGL in the marker names has been removed for clarity.

**Figure 4:**
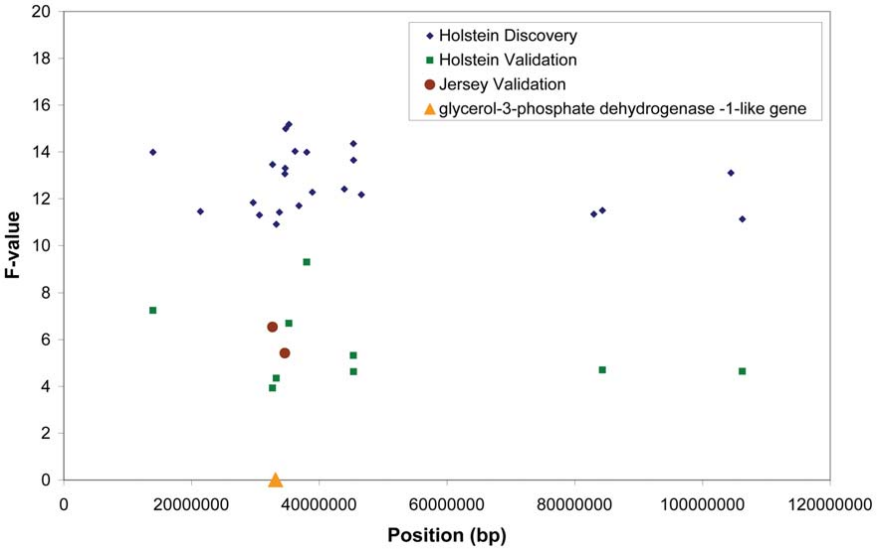
Position of significant SNPs in the discovery and validation data sets on chromosome 9. The position of the putative glycerol-3-phosphate dehydrogenase 1-like gene is indicated.

This report illustrates the power of experiments in dairy cattle. By utilising the large databases of milk production records that are maintained by the dairy industry we can estimate the genetic merit of each bull with high accuracy and consequently use a rather small number of bulls with SNP data to detect loci for such a complex trait as sensitivity of milk production to feeding level and temperature humidity index. In addition, the recent small effective population size in Holstein Friesian cattle has led to useful LD extending for considerably larger distances than in humans for example, which increase the prospects of finding associations with the marker density used in this experiment [19]–[21]. However, this leads to associations across large genomic intervals, Figure 4. These genomic intervals can be refined by using an across breed validation strategy. The extent of LD between breeds such as Holstein and Jersey is such that markers will only be validated across breeds if they are very close to the causative mutation [22], so the validation strategy we have used should map the causative mutation to a small genomic interval (eg. Figure 4). An across breed mapping strategy has been successfully used to map traits including coat pattern in dogs, a species with a similar pattern of linkage disequilibrium to cattle [23].

We investigated the list of genes and their reported functions in the region of the SNPs with validated effects in both breeds. One such SNP associated with sensitivity of milk production to THI was located on BTA29 position 48329079 bp. Of the genes in the region, the strongest candidate for harbouring a mutation affecting the trait is fibroblast growth factor 4 (48851846 bp to 48852868 bp). This gene is a regulator of mammary epithelial cells apoptosis during both morphogenesis and involution of the mammary gland. In transgenic mice over-expressing human FGF4, the most striking effect caused by FGF4 over-expression was on the remodelling of mammary tissue at the end of lactation [24]. In human testis, FGF4 expression is increased in response to increasing temperature, with a putative role in protecting germ cells [25]. FGF4 was expressed in bovine mammary gland at 90 days of lactation at a moderate level (Bovine Gene Atlas http://www.agbase.msstate.edu/bovineatlas/). While a search of dbSNP did not reveal any SNPs within exons of this gene, there is a SNP in the promoter region of the gene which warrants further testing (at 48856593 bp, ARS-BFGL-NGS-65571).

The most promising candidate for harbouring a mutation affecting sensitivity to feeding level (HTDMY_slope_) is located between the two most significant SNPs from the Jersey validation, on chromosome 9 (Table 3, Figure 4). This gene (NCBI XM_865508), between 33155321 bp and 33156376 bp, is similar to the glycerol-3-phosphate dehydrogenase-1-like gene. The translated bovine gene at this location is 88% identical to the peptide sequence of the human and bovine glycerol-3-phosphate dehydrogenase-1-like predicted proteins and 72% identical to that for bovine glycerol-3-phosphate dehydrogenase-1. Because of it's similarity to glycerol-3-phosphate dehydrogenase (G3PD), the candidate gene retains the G3PD sequence motif and might be expected to exhibit similar enzyme activity. G3PD is at the nexus of pathways for carbohydrate and phospholipd metabolism and is therefore a key enzyme for energy utilisation. In one study, a high carbohydrate diet fed for a prolonged period induced hyperglycaemia, hyperinsulinaemia, and islet hyperplasia in the mice with normal mitochondrial glycerol-3-phosphate dehydrogenase function, while mice with disrupted mitochondrial glycerol-3-phosphate dehydrogenase function did not develop these traits, but did show increased insulin sensitivity [26]. Given that insulin sensitivity differs between cows which differ in their milk production response to the level of feeding [27], we could hypothesise that a mutation in the candidate gene or it's regulatory regions alters insulin sensitivity which in turn alters the response of milk yield to the level of feeding. Two adjacent SNPs in the coding region of the gene (rs42378599 and rs42378600) have been reported in dbSNP and these warrant further testing.

In this paper we have identified and validated panels of markers to enable selection of dairy cattle for adaptation to the altered production systems that are possible under climate scenarios. For example, the validated SNPs affecting HTDMY_slope_ should be valuable to select bulls to generate daughters that will be productive at low levels of feeding, if high energy feed stuffs become increasingly scarce.
